# Retraining and assessing hand movement after stroke using the MusicGlove: comparison with conventional hand therapy and isometric grip training

**DOI:** 10.1186/1743-0003-11-76

**Published:** 2014-04-30

**Authors:** Nizan Friedman, Vicky Chan, Andrea N Reinkensmeyer, Ariel Beroukhim, Gregory J Zambrano, Mark Bachman, David J Reinkensmeyer

**Affiliations:** 1 Department of Biomedical Engineering, University of California, Irvine, USA; 2 Department of Mechanical and Aerospace Engineering, University of California, Irvine, USA; 3 Department of Electrical Engineering and Computer Science, University of California, Irvine, USA; 4 Department of Anatomy and Neurobiology, University of California, Irvine, USA; 5 Department of Physical Medicine and Rehabilitation, University of California, Irvine, USA; 6 Rehabilitation Services, Irvine Medical Center, Irvine, USA

**Keywords:** Stroke rehabilitation device, Hand, Music therapy, Video game therapy, Stroke assessment, Outcome measures

## Abstract

**Background:**

It is thought that therapy should be functional, be highly repetitive, and promote afferent input to best stimulate hand motor recovery after stroke, yet patients struggle to access such therapy. We developed the MusicGlove, an instrumented glove that requires the user to practice gripping-like movements and thumb-finger opposition to play a highly engaging, music-based, video game. The purpose of this study was to 1) compare the effect of training with MusicGlove to conventional hand therapy 2) determine if MusicGlove training was more effective than a matched form of isometric hand movement training; and 3) determine if MusicGlove game scores predict clinical outcomes.

**Methods:**

12 chronic stroke survivors with moderate hemiparesis were randomly assigned to receive MusicGlove, isometric, and conventional hand therapy in a within-subjects design. Each subject participated in six one-hour treatment sessions three times per week for two weeks, for each training type, for a total of 18 treatment sessions. A blinded rater assessed hand impairment before and after each training type and at one-month follow-up including the Box and Blocks (B & B) test as the primary outcome measure. Subjects also completed the Intrinsic Motivation Inventory (IMI).

**Results:**

Subjects improved hand function related to grasping small objects more after MusicGlove compared to conventional training, as measured by the B & B score (improvement of 3.21±3.82 vs. -0.29±2.27 blocks; P=0.010) and the 9 Hole Peg test (improvement of 2.14±2.98 vs. -0.85±1.29 pegs/minute; P=0.005). There was no significant difference between training types in the broader assessment batteries of hand function. Subjects benefited less from isometric therapy than MusicGlove training, but the difference was not significant (P>0.09). Subjects sustained improvements in hand function at a one month follow-up, and found the MusicGlove more motivating than the other two therapies, as measured by the IMI. MusicGlove games scores correlated strongly with the B & B score.

**Conclusions:**

These results support the hypothesis that hand therapy that is engaging, incorporates high numbers of repetitions of gripping and thumb-finger opposition movements, and promotes afferent input is a promising approach to improving an individual’s ability to manipulate small objects. The MusicGlove provides a simple way to access such therapy.

## Background

Hand impairment is a common condition that contributes substantially to disability in the U.S. and around the world [1]. In the case of stroke alone, it is estimated that approximately 80% of the 700,000 individuals who survive a stroke each year require hand therapy [2-4]. Other conditions that have a high incidence of hand impairment are hand and wrist trauma, high-level spinal cord injury, multiple sclerosis, traumatic brain injury, muscular dystrophies, and cerebral palsy. In all of these conditions the human motor system retains substantial capacity for plasticity, and thus intensive rehabilitation exercise reduces long-term hand impairment [5-7]. Unfortunately, there currently exist few validated technologies for at-home upper-extremity rehabilitation after a stroke. A recent systematic review of home-based upper extremity therapy analyzed only four studies, and only two of these included a self-guided intervention [8].

Therapy is limited because on-going rehabilitation exercise delivered one-on-one with a rehabilitation therapist is expensive. Gyms do not have appropriate equipment to facilitate practice of the fine motor skills needed to improve hand dexterity. Few devices are commercially available for at-home hand therapy and these are either expensive or not motivating. For example the HandMentor [9] and HandTutor [10] cost several thousand dollars, and the Amadeo (TyroMotion) is even more expensive. Virtual reality and computer gaming is promising for home-based rehabilitation because it can provide ecologically valid, intensive task specific training [11]. Systematic reviews of virtual-reality based rehabilitation delivered in the clinic indicate that is effective for arm rehabilitation, but there are fewer trials on hand rehabilitation [11,12]. Following written sheets of exercise prescribed by the therapist is therefore a fairly common low cost approach to hand therapy, but this approach often lacks in intensity, repetition, and motivational value—factors thought to be important for maximizing hand movement recovery [4-7,13]. Without guidance and a motivating rehabilitation regimen, individuals cease practice of their affected hand and do not recover to their full potential [2,3,14,15].

Participating in music is a promising avenue for therapy because it is motivating, challenging, sensory-rich, and repetitive [16]. Further, participating in music after a stroke can induce plastic changes in the motor cortex as well as increase attention span, neuropsychological scores, cognitive functioning and well-being [17-24]. fMRI studies show that motor and auditory temporal processing are coupled during the act of listening, meaning the motor system is responsive to the auditory system [25-27].

Recognizing the potential benefits that music therapy provides, several devices for music-based therapy have been developed to focus on movement repetition and auditory feedback [16,20]. Adamovich developed a virtual piano trainer to retrain finger dexterity after stroke using a haptic device (CyberGrasp) worn over a dataglove (CyberGlove) [28]. Alten Muller and Schneider developed a customized electronic keyboard and drum pad designed to train gross and fine hand movement [16]. Although these devices promote movement using music, they are not focused on training hand movements used in activities of daily living such as key-pinch grip and pincer grasp. Based on the specificity of learning hypothesis in motor behavior, which holds that motor learning is most effective when practice sessions include movement conditions that closely resemble those required during performance of the task [29-31], training functional movements may be more beneficial to regaining motor function.

Objectively measuring hand use during therapy can be beneficial in providing effective rehabilitation. Quantitative feedback about movement performance can improve recovery of motor function in people with stroke [32]. It also enables users to track improvements in hand use, and provides an objective, unbiased, account of a patient’s movement practice.

**Figure 1:**
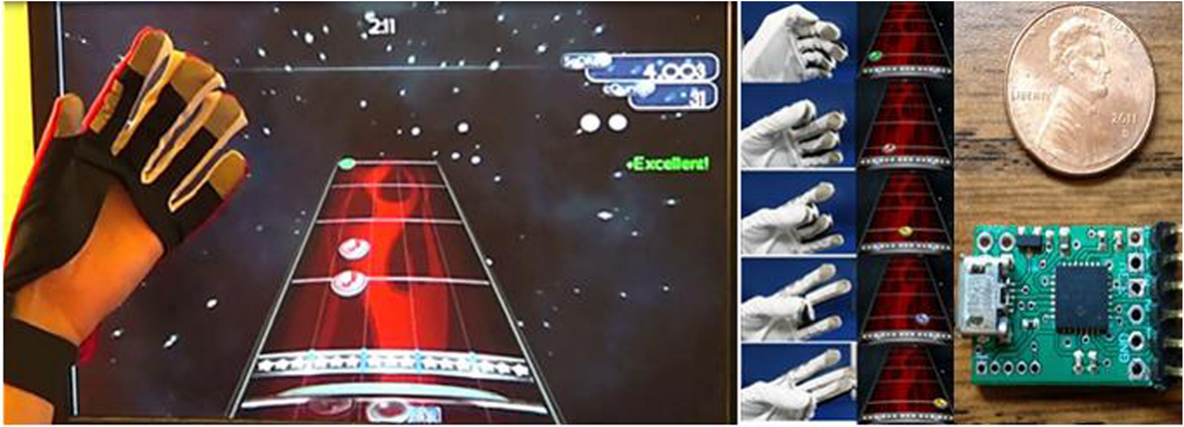
**The MusicGlove is a sensorized glove that requires the user to make functional gripping movements to play a customized version of the open source music game called Frets on Fire, which was inspired by Guitar Hero.** When the scrolling notes on the screen reach the white marker at the bottom, the user must make a specific grip associated with each note. The five grip types associated with each notes are shown (middle). When the electrical lead on the thumb touches one of the other five leads on the fingers, a custom-made controller (right) sends event data to a computer through an HID USB protocol.

We developed the MusicGlove, a music-based rehabilitation device to help people regain hand function both in the clinic and at home [33,34] (Figure 1). It is an instrumented glove that requires the user to practice functional gripping movements to play a customized version of an open-source music game called Frets on Fire (FOF) inspired by the third most popular video game franchise to date, Guitar Hero. When the user touches the thumb lead on one of the five electrical leads on the fingertips or lateral aspect of the index finger, the device sends a signal to the computer through the USB port using a custom-made controller (Figure 1, right). The leads are positioned so as to require functional grips such as pincer grip or key pinch grip. In the computer game, colored notes scroll down the screen on five distinct frets. When the notes reach the bottom of the screen, the user must touch one of the respective leads on the glove with her thumb within a specifiable time window (Figure 1, left). Hitting the note causes it to explode, and increases the music volume, while missing the note decreases the volume. Correct notes are logged and displayed in a summary at the end of the game, providing a quantitative assessment of hand motor performance, including which grip the user is best at completing and how accurately the user completes each target gripping movement in the desired time window (e.g. late or early).

We previously conducted a usability study at the University of California, Irvine with 10 participants with chronic stroke. We found that the MusicGlove can be donned and used by people with severe hand impairment quantified by a Box and Block score of 7 (this test measures the number of blocks that subjects transport in a minute and a normal score is about 60) [33]. Further, the MusicGlove-based assessments of hand function were correlated with standard clinical evaluations (i.e. Box and Block score), suggesting the device provides self-measurable, quantitative feedback to users, clinicians, and caregivers relevant to rehabilitation progress. We also found that the addition of music to hand movement practice in a single training session significantly improved both objective measures of hand motor performance during training and self-ratings of motivation for training. In a questionnaire, the majority of participants stated that the device was a motivating tool for therapy, and that they would like to continue using it for rehabilitation.

**Figure 2:**
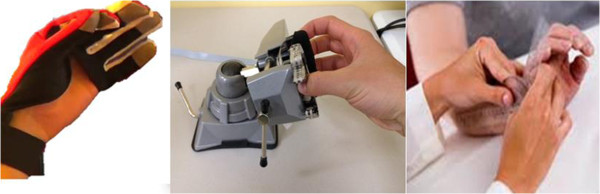
**The three hand therapies tested; MusicGlove (left), IsoTrainer (middle) – a device that requires isometric gripping and conventional tabletop hand exercises with an experienced rehabilitation therapist (right).** Training consisted of 6, one-hour long training sessions over the course of two weeks for each treatment type.

The first aim of this pilot study was to test whether training with the MusicGlove would improve hand motor function. We hypothesized that following multiple training sessions, the MusicGlove would significantly improve hand motor control in people with a chronic stroke when compared to conventional tabletop exercises. In this study, we also used the MusicGlove to study the role of proprioceptive input in facilitating hand motor recovery. The second aim was that the more propriocpetively-rich movement training associated with the MusicGlove would produce larger improvements in hand motor control, compared to a matched form of isometric movement training, in which the fingers statically gripped a stationary object (the IsoTrainer, Figure 2, middle) and played the same music-based game. An implicit rationale that has been used to support the development and application of robotics technology for movement rehabilitation is that assisting individuals in completing movement will enhance somatosensory input, facilitating sensory motor recovery through Hebbian-like mechanisms [35,36]. Although the MusicGlove is not robotic, use of it generates proprioceptive input associated with the dynamic finger movement it requires, as well as the making and breaking of finger-to-thumb contacts. A third aim was to study how game score performance correlated with the primary outcome measure, the Box and Block score, since an important issue in rehabilitation science and practice is to improve objectivity, sensitivity, and ease of measurement of upper extremity outcomes.

## Methods

### Subjects

A total of twelve adult stroke survivors were recruited through local hospitals, stroke support groups in Orange County, CA, and a database of people who had completed previous stroke studies at the university. The study was conducted at the Sue and Bill Gross Stem Cell Research Center at the University of California in Irvine. All participants suffered from a single ischemic or hemorrhagic stroke and were at least six months post-stroke at the time of their enrollment into the program. All participants demonstrated mild to moderate upper extremity impairment as defined by the upper extremity Fugl-Meyer score (range 34 – 62) and mild to severe hand impairment as defined by the upper extremity Box and Block (B & B) test (range of 1–55) [37,38]. Exclusion criteria included significant pain of the affected upper extremity, severe tone in the affected upper extremity that affects movement, severe loss of sensation of the affected upper extremity, concurrent severe medical problems, cognitive dysfunction to an extent that would interfere with therapy participation, visual deficits, severe neglect or apraxia, and enrollment in other upper-extremity therapy studies. All subjects provided written consent, and all procedures were approved by the Institutional Review Board of the University of California in Irvine. Table 1 shows demographic information for participants.

**Table 1:** Subject demographics

**Gender**	**7 males, 5 females**
Age	57 +/- 30.5 SD, range 19–80
Months post stroke	34.6 +/- 32.5 SD, range 8–63
Side of Hemiparesis	4 left, 8 right
Type of stroke	6 ischemic
	4 hemorrhagic
	2 unsure

### Devices

The MusicGlove was described in the Introduction section and further details can be found in [34]. To study the effect of isometric training with the hand, we developed the IsoTrainer, an isometric version of the MusicGlove device (Figure 2, left). To use the device, the person grips force sensors (Flexiforce) located on two neoprene covered acrylic boards located on opposing sides. When the user squeezes both the sensor on the thumb and one of the other respective sensors with digits 2–5 with roughly 10 N of force, an event is sent to a custom-made USB controller that transmits HID USB commands to the same Frets-on-Fire-like game used with the MusicGlove. A 360 degree rotating base accommodates multiple hand orientations.

### Assignment and intervention

We compared three types of motor hand therapies consisting of training with the MusicGlove (Figure 2, left), IsoTrainer (Figure 2, middle) and conventional tabletop exercises (control) (Figure 2, right). The duration of training was matched for each type of therapy. We used a within-subject study design where each subject participated in six one-hour treatment sessions, approximately three times per week for two weeks, for each training type, for a total of 18 one-hour treatment sessions. We then analyzed how much motor control improved after the six one hour sessions for each training type. All training sessions were supervised by the same trained physical therapist who assisted the subjects in completing the training.

Subjects were randomly assigned to groups that experienced the MusicGlove, IsoTrainer, and control training in different orders. To ensure a match in impairment severity between groups, subjects were first blocked by the B & B assessment (0–30, 30–60) and then randomized using a table generated by a statistician. The treating therapists and subjects were blinded to group assignment until each subject was consented and enrolled in the study.

**Figure 3:**
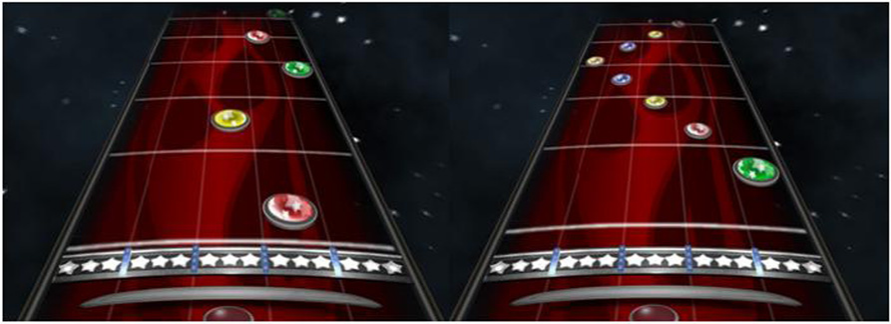
**We developed two assessments in the music game that were administered at the beginning and end of each training session.** The Dexterity test (left) contains notes on frets 1–3 that appear in a random sequence. The Speed test (right) contains notes on frets 1–5 that appear in an orderly sequence. In both assessments, notes gradually become closer together in time as the song progresses.

During the MusicGlove intervention, participants first donned a fitted glove on the affected hand and played the tutorial song, for a duration of 1 minute and 10 seconds, in the FOF game to practice using the device. Subjects then performed the Dexterity assessment song containing 42 notes for a duration of 1 minute and 26 seconds (Figure 3, left), and the Speed assessment song containing 81 notes for a duration of 1 minute and 50 seconds (Figure 3, right); these MusicGlove-based assessments are described in more detail below. They then played twelve songs with the MusicGlove, taking roughly 45 minutes to complete and replayed the Speed and Dexterity assessments at the end of treatment. At the conclusion of each song, the therapist logged the total% notes hit and% notes hit on each finger (this information is displayed at the end of the song). In total, thus, subjects were instructed by the game to move a total of 1420 times per session.

During the IsoTrainer intervention, participants were fitted to the device and performed the same Speed and Dexterity assessments before and after each training session. They completed the number of songs as with MusicGlove therapy in the same sequence. Performance scores (same as with MusicGlove therapy) were logged at the end of each song.

During the control intervention participants followed a series of conventional tabletop exercises (used at therapy clinics at the University of California in Irvine and Ohio University) consisting of passive range-of-motion stretches, active range-of-motion exercises, isometric strengthening exercises, and functional gripping practice for the hand. Time spent on each movement and number of total repetitions in a session was not controlled; only the total therapy time per session was controlled. If participants completed all prescribed exercises, they were asked to start again at the beginning until the 45 minute training session was complete. During passive stretches, subjects used their unaffected limb to move their affected hand. During active exercises, subjects practiced using their affected wrist and hand without the help of the unaffected hand. During isometric training, subjects performed active movements while using their unaffected hand to keep the affected hand in a static position. Subjects performed passive exercises for roughly 15-20% of the duration of each session. Functional gripping practice included touching the thumb to each fingertip, making a pincer grasp, power grasp, practicing thumb opposition, picking up coins, and picking up a pencil. Refer to the Additional file 1 entitled “Assessment Procedures” for more information on the administered table-top exercises.

Subjects were assessed at one and two weeks prior to the study, after the completion of the six treatment sessions, and at a one-month follow-up assessment. A single blinded evaluator who is an experienced occupational therapist performed all of the clinical assessments during all testing sessions. The primary outcome measure was the Box and Block Test (B & B), which measures how many blocks a subject can pick up and place in a box in 60 seconds. Other outcome measures included the following: 1.) the arm motor section of the Fugl-Meyer score (AMFM) [39], which measures motor function of the subjects’ hemiparetic arm; 2.) the Action Research Arm Test (ARAT) [40], which measures the time to complete various manipulation activities with the hands; 3.) the Wolf Motor Function test (WMFT) [41], which measures the performance of a list of functional activities with the arm; and 4.) the 9-hole peg test (NHPT) [4,42-44], which measures how many pegs a subject can put in and remove from the holes in a fixed amount of time (reported in pegs/minute).

We measured hand grip strength using a hydraulic hand dynamometer (Jamar 5030 J1) and pinch strength between the thumb and digits 2–5 using a hydraulic pinch gauge (Jamar 7498–05). Participants were asked to sit upright with their shoulder at a neutral position, their elbow flexed to 90 degrees, and their forearm at a neural position. Participants were then asked to squeeze as hard as possible and then release. This procedure was repeated three times for each grip with both unaffected and affected hand. The average of the three measurements for each grip was reported.

We chose the Box and Blocks as the primary outcome measure because it quantifies an important skill – grasp and transport, is simple and quick to administer, and is more objective than FM, ARAT and WMFT because it depends less on the semi-subjective rating of the evaluator. Box and Blocks has excellent reliability as well, and is correlated with FM and ARAT [35]. FM likely suffers from a ceiling effect for subjects with higher scores on this test, such as the ones we tested [45].

Following the six training sessions with each treatment type, participants completed selected items from the Intrinsic Motivation Inventory (IMI), a reliable and valid survey used to quantify motivation [46], and a user satisfaction questionnaire. For the IMI, we selected 13 of its 44 questions that were distributed into five subscales categorized as effort/importance, pressure/tension, perceived competence, value/usefulness, and interest/enjoyment. The selection of specific items from the IMI was suggested as a valid approach by the original developers of this scale [47]; subsets of IMI items analyzed together have been shown to be reliable [46].

We also used the results from the Dexterity and Speed tests described in Figure 3 as outcome measures. The names of these tests were chosen somewhat arbitrarily, but the Dexterity test was designed to evaluate how well subjects could respond to an unpredictable sequence of notes that continuously sped up. This test used only 3 notes in order to minimize the cognitive processing needed to complete the task. The Speed test in contrast was designed to test how well subjects could complete a predictable sequence of notes that sped up, and tested all finger-thumb pairs. Presenting the notes in a predictable way minimized the need for cognitive processing in this test; the core requirement was to move the fingers in an increasingly fast, but known, pattern.

### Statistical analysis

A Student’s t-test showed no significant improvement between assessments one and two (administered pre-training); these assessments were averaged to establish a baseline. Outcome measures did not deviate significantly from normality (Kolmogorov-Smirnov test, P > .05). Therefore repeated measures analyses of variances (ANOVAs) were used with training type as the within-subjects repeated factor. A Greenhouse-Geisser adjustment was used when sphericity was violated (P < .05). One tailed, paired Student’s t-tests were then applied to evaluate whether there were differences in outcomes among the three training conditions, given our starting hypotheses that MusicGlove therapy would be more beneficial than IsoTrainer therapy and conventional therapy. The omnibus Friedman test and post-hoc Wilcoxon signed-rank test were used to compare user satisfaction survey results and the IMI for the three training types. A P value of < .05 was used as statistically significant, and a Bonferroni correction was applied to all post-hoc outcome measure tests and survey results to account for family-wise error (P = 0.05/3). To account for floor effects, 9HPT score was calculated as pegs per minute [48].

## Results

We measured how a matched duration of training (six 45 minute sessions) with the MusicGlove, IsoTrainer, or standard tabletop exercises (control) affected hand motor control in a within-subjects design.

### Clinical outcome measures

**Figure 4:**
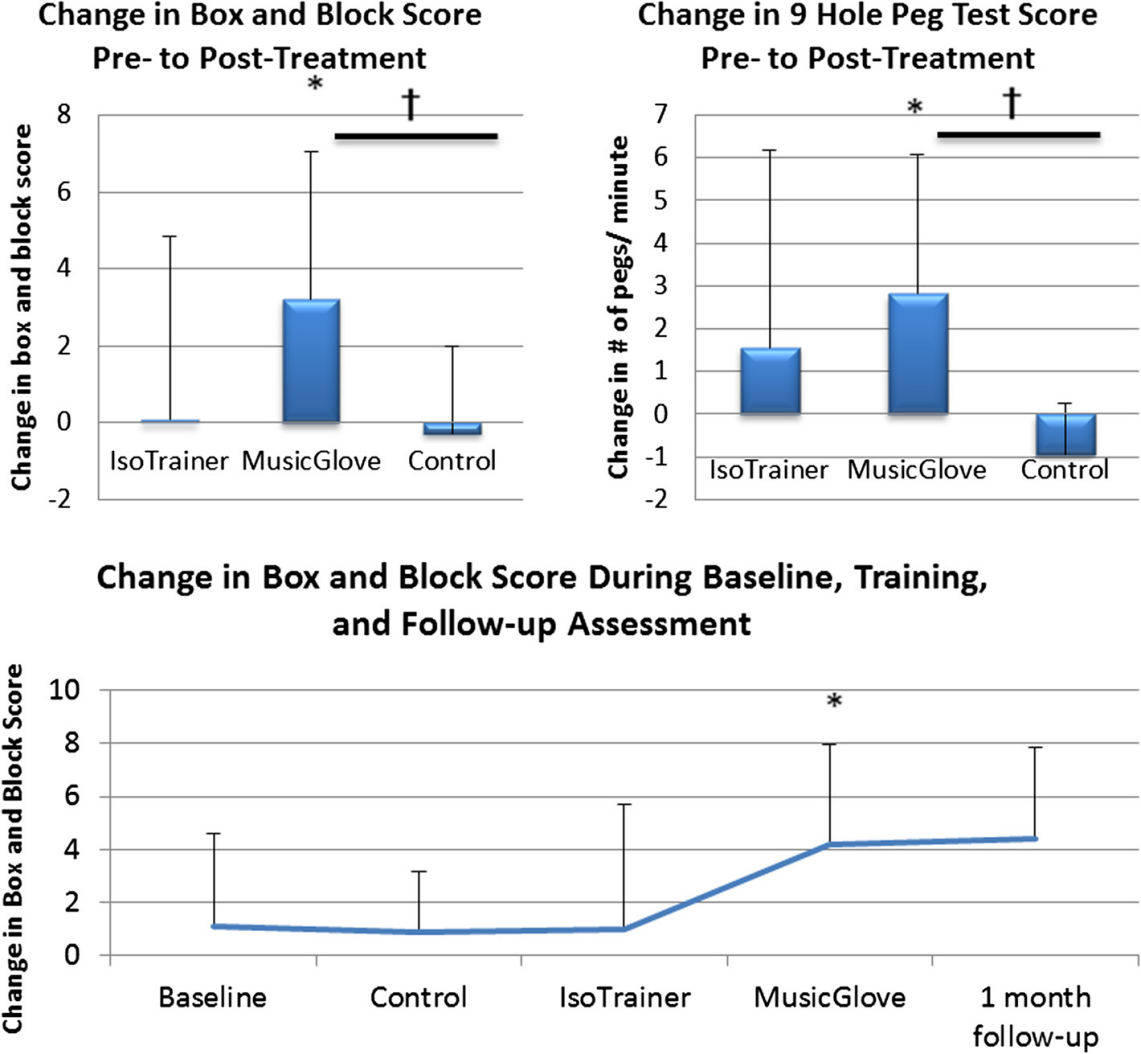
**After two weeks of training with the MusicGlove, subjects demonstrated significant improvement in hand function pre- to post-assessment as measured by the Box and Block score (top left plot) and the 9 Hole Peg Test (top right plot).** Participants also showed a significant improvement in these assessments while using the MusicGlove compared to the table-top exercises guided by a physical therapist (control). The bottom plot shows cumulative gains over the course of the study. Baseline gains represent the difference between assessment 2 and assessment 1. One month follow-up represents the difference between the final administered assessment and the second to last assessment following the final treatment. Treatment groups were randomized but are shown as ordered for graphical purposes. P < 0.017 is considered significant with an applied Bonferroni correction. *Significant improvement pre- to post-treatment. ^†^Significant improvement between the two training types.

The baseline values and changes in assessments after six training sessions are summarized in Table 2. Subjects demonstrated significantly greater improvement with two weeks of training with the MusicGlove over two weeks of control training in B & B score, the primary outcome measure (P = 0.010). MusicGlove training significantly improved B & B score pre- to post-training (P = 0.009). The MusicGlove, IsoTrainer, and control treatments improved B & B score an average of 3.21 (±3.82 SD), 0.083 (±4.75 SD), and -0.29 (±2.27 SD) blocks respectively (Figure 4, top left). Subjects also demonstrated significant improvement with MusicGlove training over control training in the NHPT assessment, (t (11) = 3.06, P = 0.005), improving on average 2.14 (±2.98 SD), 1.47(±4.55 SD), and -0.85 (±1.29 SD), pegs per minute respectively, with MusicGlove, IsoTrainer, and control training (Figure 4, top right).

**Table 2:** **Outcome measures overview**^**a**^

	**Baseline measure**	**Change in outcome measure pre- to post-training**			**Repeated measures ANOVA**	**P Between Training types**			**P pre-to-post training**		
**Outcome Measure**		**MGL**	**TTE**	**ITR**		**MGL vs TTE**	**MGL vs ITR**	**ITR vs TTE**	**MGL**	**TTE**	**ITR**
Box and Block Test (blocks/min)	28.4 ± 15.8	3.21 ± 3.82	-.29 ± 2.27	.083 ± 4.75	.071	.010^b^	.092	.440	.009^b^	.384	.477
Arm Motor Fugl-Meyer (out of 66)	53.2 ± 7.29	.875 ± 3.19	.750 ± 2.14	1.83 ± 2.37	.571	.460	.220	.184	.191	.134	.013
Wolf Motor Score	2.87 ± .290	.101 ± .141	.040 ± .079	.056 ± .127	.466	.155	.269	.350	.014^b^	.061	.088
Wolf Motor Time (seconds)	13.4 ± 11.5	-1.58 ± 3.13	-1.38 ± 3.05	-2.29 ± 3.84	3.83	.447	.253	.274	.061	.081	.037
Action Research Arm Test	38.1 ± 14.8	.875 ± 2.85	.167 ± 4.12	1.41 ± 3.44	.223	.344	.367	.277	.166	.448	.100
9-Hole Peg (Pegs-minute)	7.10 ± 6.45	2.14 ± 2.98	-.855 ± 1.29	1.47 ± 4.55	0.011^b^	.005^b^	.370	.069	.015^b^	.026	.153
Key pinch (kg force)	3.90 ± 1.85	0.417 ± 2.17	-.200 ± 3.41	.754 ± 1.06	.646	.341	.353	.203	.269	.425	.019
Index pincer (kg force)	1.55 ± .880	.146 ± .463	.092 ± .672	.200 ± .347	.881	.419	.373	.345	.160	.330	.041
Middle pincer (kg force)	1.68 ± 1.02	-.007 ± .686	.154 ± .668	.344 ± .712	.714	.322	.134	.238	.487	.230	.069
Ring pincer (kg force)	1.09 ± .871	.039 ± .414	.183 ± .783	.311 ± .763	.644	.340	.135	.380	.380	.227	.102
Little pincer (kg force)	.601 ± .641	-.108 ± .240	.100 ± .537	.175 ± .667	.899	.175	.100	.415	.081	.275	.201

*Abbreviations:MGL*, MusicGlove; *ITR*, IsoTrainer; *TTE*, Tabletop exercises (control).

^a^Values are given as means ± standard deviations.

^b^P < .05/3.

Figure 4, bottom, shows a plot of cumulative gains over the course of the study due to all three training types (i.e. over all 18 sessions) and illustrates that training benefits, which mostly occurred during the MusicGlove portion of training, persisted at the one month follow-up. There was no difference between training types in the assessment batteries that assessed a broader range of hand function – the Wolf Motor Function Test and Action Research Arm Test -- although each training type significantly or nearly significantly improved in the WMFT (Table 2).

### Survey results

**Figure 5:**
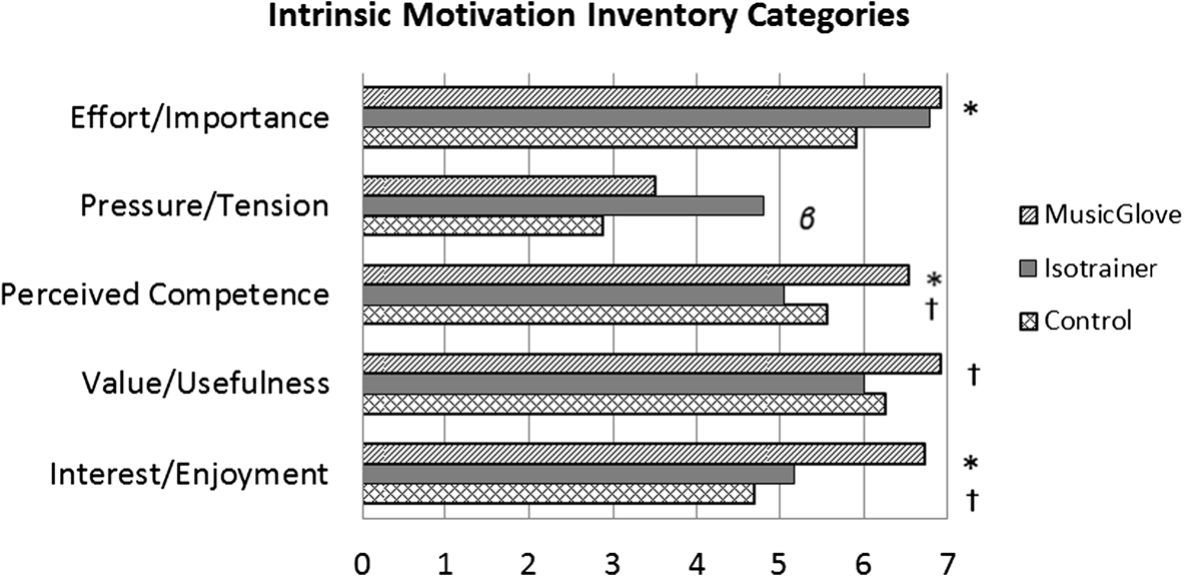
**Results of the Intrinsic Motivation Inventory survey given after six training sessions with each training type.** Significance measured by post-hoc Wilcoxon signed-rank test. P < 0.017 is considered significant with an applied Bonferroni correction. *Significant difference between MusicGlove and control. ^†^Significant difference between MusicGlove and IsoTrainer. ^β^Significant difference between IsoTrainer and control.

A user satisfaction questionnaire and a subset of the Intrinsic Motivation Inventory (IMI) [46] were administered following each treatment (Figure 5). Participants reported: MusicGlove was significantly more effortful and important than control (P = 0.008); IsoTrainer caused significantly more tension than control (P = 0.001); feeling significantly more competent with using MusicGlove over control (P = 0.002) and MusicGlove over IsoTrainer (P < 0.001); MusicGlove was more interesting than control (P < 0.001), and MusicGlove more interesting than IsoTrainer (P < 0.001).

**Figure 6:**
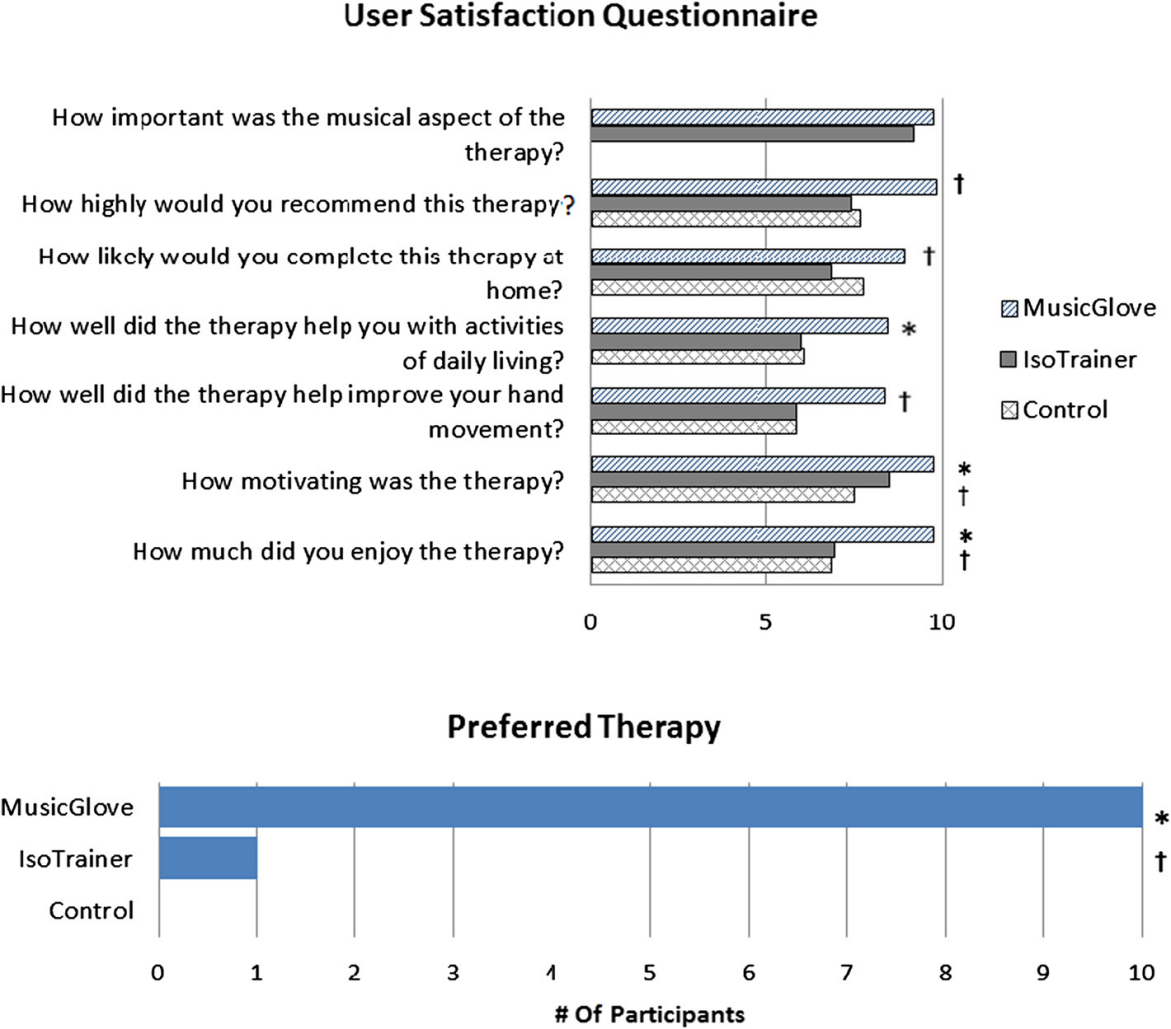
**Results of the user satisfaction survey given after all three training types were completed.** Significance measured by post-hoc Wilcoxon signed-rank test. P < 0.017 is considered significant with an applied Bonferroni correction. *Significant difference between MusicGlove and control. ^†^Significant difference between MusicGlove and IsoTrainer. ^β^Significant difference between IsoTrainer and control.

After all 18 sessions, participants also completed a user satisfaction questionnaire tailored to the study (Figure 6). Subjects reported the musical aspect of therapy was highly important (mean = 9.5/10); recommended MusicGlove over IsoTrainer (P = 0.015); were more likely to complete MusicGlove over IsoTrainer therapy at home (P = 0.002); reported MusicGlove helped more than control with activities of daily living; reported MusicGlove helped more than IsoTrainer (P = 0.004) for improving hand movement; reported MusicGlove was more motivating than control (P = 0.008) and IsoTrainer (P = 0.008) for improving hand movement; enjoyed MusicGlove more than control (P = 0.016) and IsoTrainer (P = 0.016); and found the musical aspect of the therapy very important for both the MusicGlove and IsoTrainer (average = 9.5). 11 out of 12 participants preferred MusicGlove therapy over IsoTrainer, and control (P < 0.001).

### MusicGlove assessments

An important issue in rehabilitation science and practice is to improve objectivity, sensitivity, and ease of measurement of clinical outcomes. We therefore studied how the overall game score for the MusicGlove correlated with the primary clinical outcome measure, the Box and Block score.

### Relationship between MusicGlove game score and primary clinical outcome measure (Box and Block Score)

**Figure 7:**
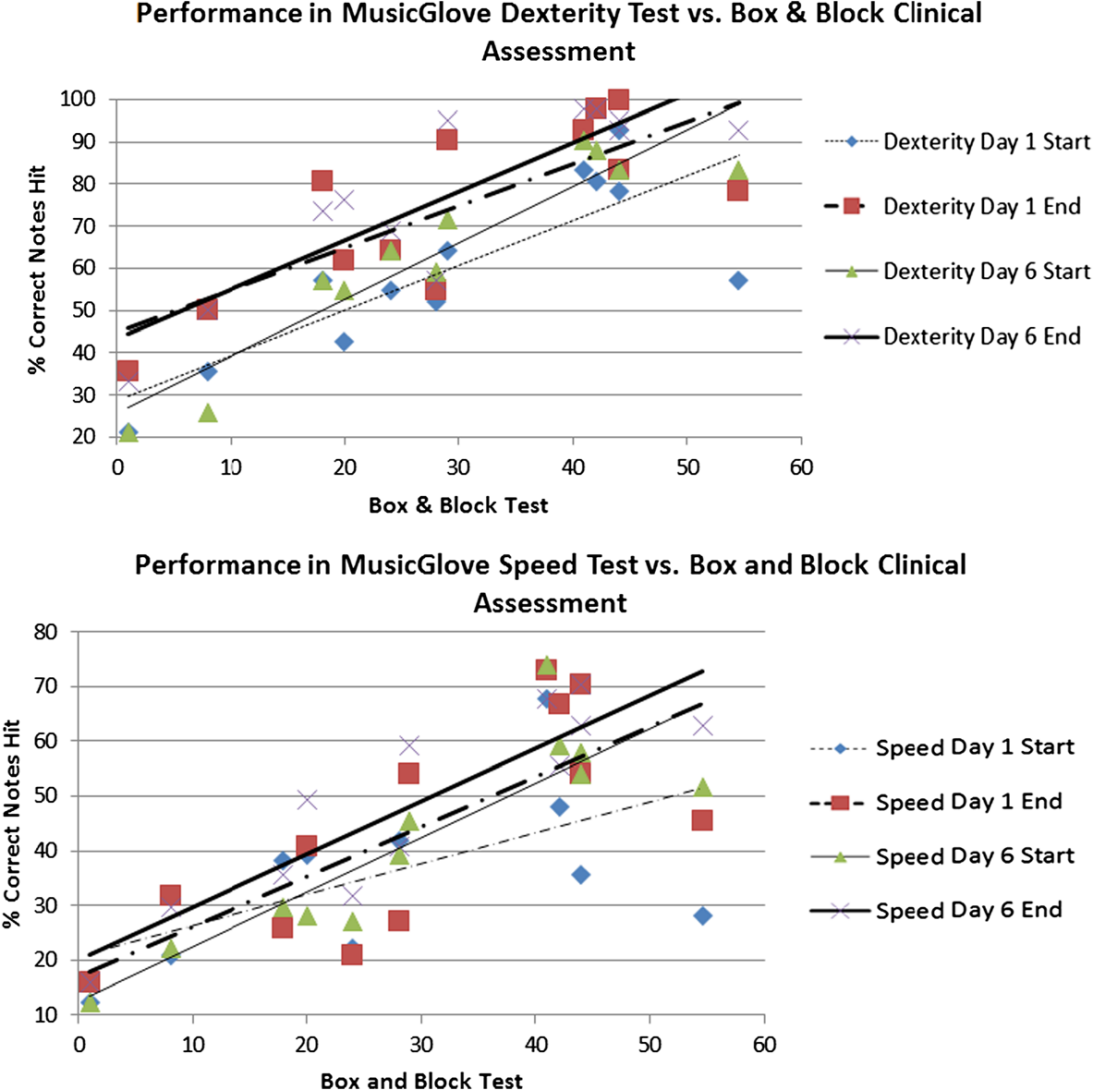
**Comparison of the performance of the Dexterity test (top) and Speed test (bottom) using the MusicGlove, and the B & B clinical score.** Thin lines represent game performance at the start of each day, thick lines represent a game performance at the end of each day, dashed lines represent performance during day 1, and solid lines represent performance at 6. The MusicGlove score significantly predicted B & B score in all 8 conditions.

Participants used the MusicGlove to play the Speed test and Dexterity test at the start and end of each training session for the 6 training sessions they trained with the MusicGlove. We compared the participants’ percent of total notes hit for both tests with B & B score at the start and end of day 1 and day 6 (Figure 7). We found that participants with a B & B score of 1 or more could use the MusicGlove to correctly hit at least 20% of the notes. A linear regression was preferred over an exponential fit or power fit based on the Akaike Information Criterion [49]. There was a significant linear relationship between all combinations of FOF scores and B & B scores (Table 3). Participants showed a significant improvement of 2.7% (±3.1 SD) in FOF score from the start of day 1 to the start of day 6 for the Dexterity test (t (11) = 1.87, P = 0.044). Participants improved on average: 6.7% (±7.2 SD) in the Speed test from start to end of day 6 (t (11) = 3.07, P = 0.005), 7.3% (±4.3 SD) in the Dexterity test from start to end of day 1 (t (11) = 4.84, P < 0.001), and 6.37% (±3.9 SD) in the Dexterity test from start to end of day 6 (t (11) = 5.38, P < 0.001).

**Table 3:** Relationship between MusicGlove test scores and Box & Block test score

	**P**	**r**^**2**^
Dexterity test day 1 start	0.001	0.67
Dexterity test day 1 end	0.003	0.88
Dexterity test day 6 start	0.001	0.61
Dexterity test day 6 start	<0.001	0.75
Speed test day 1 start	0.046	0.45
Speed test day 1 end	0.004	0.61
Speed test day 6 start	<0.001	0.78
Speed test day 6 end	<0.001	0.8

### Relationship between MusicGlove game score for the five grip types and Box and Block Score

**Figure 8:**
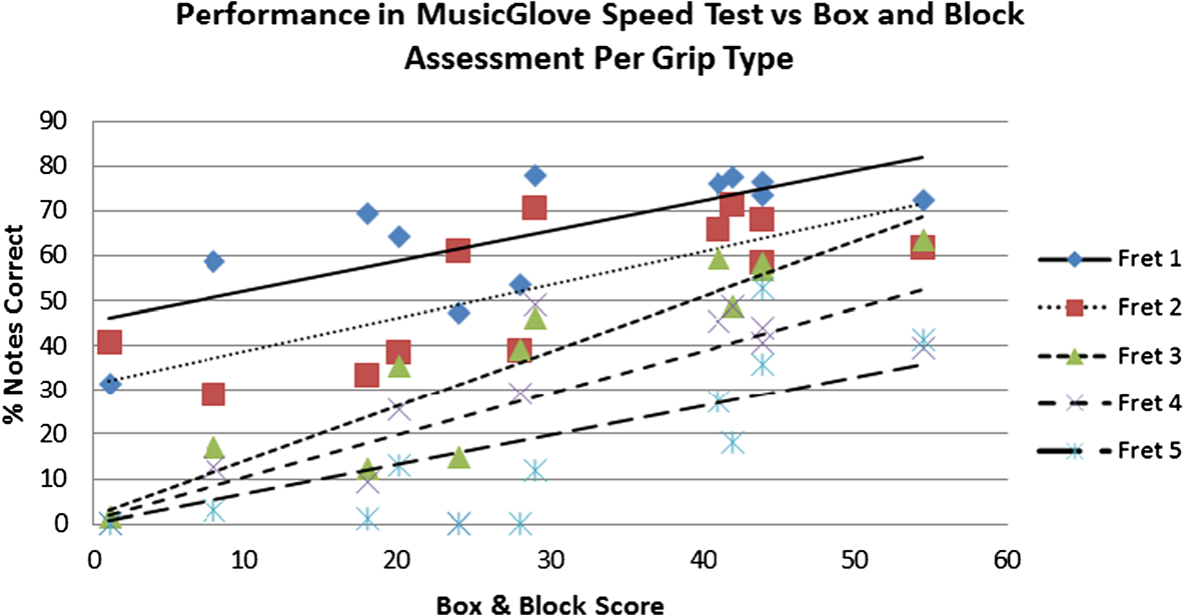
**Comparison of the% notes hit correct during the Speed assessment with B & B score.** All five grip types were strongly correlated with B & B score. Data points represent the average of each participant’s Speed assessment performance over the course of the six training sessions.

We compared participant’s performance (i.e.% notes correct) of key-pinch grip (fret 1), pincer grip with index finger (fret 2), pincer grip with middle finger (fret 3), pincer grip with ring finger (fret 4), and pincer grip with little finger (fret 5) with B & B score using the Speed assessment test (Figure 8). A linear regression was again preferred over an exponential or power fit based on the Akaike Information Criterion. There was a linear relationship between each of the five grip types exercised using the MusicGlove and the B & B score: P fret 1 = 0.006, r^2^ = 0.56; P fret 2 = 0.007, r^2^ = 0.56; P fret 3 = 0.012, r^2^ = 0.86; P fret 4 = 0.009, r^2^ = 0.65; P fret 5 = 0.009, r^2^ = 0.62). 4 out of 12 participants could not perform a pincer grip with the small finger (fret 5).

### Relationship of grip type and MusicGlove game performance

**Figure 9:**
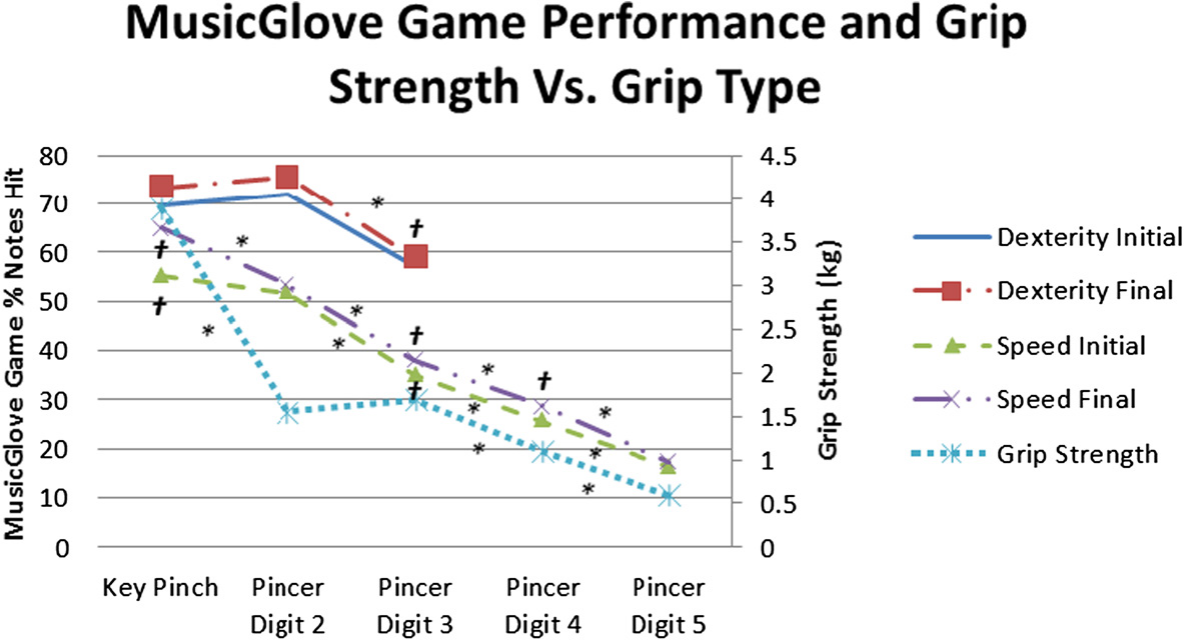
**MusicGlove Dexterity assessment and Speed assessment for the start (initial) and end (final) of each day of training (primary y-axis) along with grip strength measured by a dynamometer post-training (secondary y-axis) are compared with the five grip types used with the MusicGlove.** Data points represent the average of all participants’ scores over the 6 training sessions. *Significant difference between adjacent grip types. ^†^Significant linear relationship between game score and grip strength.

We compared the total % notes hit (primary y-axis) in the Dexterity test and Speed test administered at the beginning and end of training averaged across all 6 training sessions and grip types (Figure 9). We also compared the grip strength measured post-treatment with a dynamometer for each grip type (secondary y-axis). Participants on average performed better in the Dexterity test (average = 69.3% ±21.5%) than the Speed test (average = 40.4% ± 15.1%), suggesting that the Dexterity test was overall less difficult. Participants overall performed better with key pinch grip and pincer grip over the other grip types with the index finger in both MusicGlove Speed and Dexterity assessment. Participants performed sequentially worse in frets 1–5 in the Speed assessment administered at the end of each training session (P fret 1 and 2 = 0.007, P fret 2 and 3 = 0.003, P fret 3 and 4 = 0.009, P fret 4 and 5 = 0.009). Participants performed sequentially worse for grip types 3–5 with the grip strength assessment (pincer digit 3 and pincer digit 4, P < 0.001, pincer digit 4 and pincer digit 5, P = 0.002). The average gain for the first session to the last session of training with the MusicGlove, average across all grip types was 3.0% (±2.4% SD, P = 0.07) for the Dexterity test and 3.6% (±2.0% SD, 0.035, P = 0.04) the Speed test.

## Discussion

We developed the MusicGlove device to motivate people to complete a high number of functional gripping movements using a modified version of a popular music-based computer game. We tested this device against tabletop exercises guided by a rehabilitation therapist, and an isometric grip training protocol that used the same music-based game. First we will discuss the comparison of the MusicGlove with conventional therapy and then the comparison with the isometric training protocol. We will then discuss the use of the MusicGlove as an assessment tool.

### Comparing outcomes of MusicGlove and conventional therapy

This study examined whether training with the MusicGlove improved hand motor function more than conventional tabletop exercises. We found that participants training with the MusicGlove significantly improved hand function related to grasping small objects (measured by B & B score and 9HPT) pre- to post-treatment, and to a greater extent when compared to the conventional exercise. Participants did not experience such gains on average after conventional therapy; this finding is not unique to this study and thus emphasizes the need to develop new ways to deliver effective rehabilitation for people with chronic stroke [50-52]. Improvement in fine gripping function with the MusicGlove lead to qualitative, self-reported functional gains such as fastening a bra, double-clicking a mouse, controlling chopsticks, tying shoes, washing dishes, using a remote control, and using the restroom independently. Further, the IMI survey and user satisfaction questionnaire indicated that participants overwhelmingly found training with the MusicGlove paradigm more interesting, useful, and motivating.

These findings are compatible with previous findings of effectiveness of robotic hand therapy (reviewed in [53]) and virtual reality-based therapy (reviewed in [12]): technology based rehabilitation is promising for improving motivation and quantification of therapy and its outcomes, and enables small amounts of functional benefit. The uniqueness of the MusicGlove is that it is a simple, safe, easy-to-use technology, compared, for example, to robotic devices, and it incorporates a time-proved, very popular, motivating game, compared, for example, to most custom-built virtual reality games for therapy.

There are several limitations of the study that should be considered. First, the sample size was small. Second, the within-subjects design precludes the possibility of definitively attributing the long term gains we measured at one month to the MusicGlove portion of the training protocol. Third, as stated above, the improvement in outcomes were small. To our knowledge, the minimally clinically important difference in the B & B score has not been assessed for chronic stroke subjects, but a 3 point improvement is clearly a small effect. The participants reported practical benefits to their home life due to the overall training experience, but at present, the results should be viewed as promising initial findings that must be improved upon. Fourth, we randomized participants to different orders of intervention to mitigate order effects but did not test for order effects due to the limited sample size. We did not account for possible cumulative effects of intervention when statistically analyzing outcome measures and we did not test retention after each intervention to gain more insight into learning effects. These are limitations that should be addressed in future, larger studies.

We hypothesize that the MusicGlove paradigm was more effective at improving performance of fine gripping because it motivated a large number of goal-directed functional movements of the thumb and fingers (a likelihood we nonetheless could not objectively evaluate in the present study because we did not count thumb or finger movements in the conventional therapy), and integrated sensory-rich visual and auditory feedback that motivated high effort levels, factors thought to be important for promoting recovery that have been emphasized in previous studies of virtual reality-based exercises [12]. Future studies should examine whether training with the MusicGlove paradigm leads to larger functional gains per unit of therapy time and higher patient compliance than conventional therapy.

The movements practiced with the MusicGlove emphasized thumb mobility, although some movement of the fingers was necessary as well. The emphasis on thumb mobility may explain why we observed the greater improvements in the outcome measures that tested fine gripping tasks (i.e. B & B and NHPT), but did not observe greater improvements in the outcomes that measured a broader set of constructs related to hand and upper extremity function (ARAT and Wolf).

### Comparing outcomes of MusicGlove and IsoTrainer therapy

A secondary aim was to test whether dynamic movement training associated with the MusicGlove would produce larger improvements in hand motor control compared to a matched form of isometric movement training with the IsoTrainer. We found that participants on average improved more with the MusicGlove than the IsoTrainer in the B & B test although this improvement only approached significance. There was also a trend towards increased grip strength for the IsoTrainer (Table 2), but this did not manifest as a gain in hand function. We speculate that functional gains for people with this level of starting hand impairment are more readily attained through training that incorporates movement of the thumb and fingers, perhaps because of the greater proprioceptive input such movement delivers, a possibility that should be studied in future research.

The user satisfaction questionnaire results given after subjects had experienced all three training types revealed that 11 out of 12 participants preferred the MusicGlove, and the IMI and survey results suggest that subjects found it to be a more effective, motivating, and useful tool for rehabilitation. Interestingly, participants experienced significant gripping gains pre- to post treatment and overwhelmingly preferred MusicGlove treatment even though participants played the same computer game and the same songs with both devices. This may be attributable to the MusicGlove being easier to use than the IsoTrainer, and incorporating dynamic movement in the training. Interestingly, the one participant (out of twelve) who preferred the IsoTrainer over the MusicGlove was also the most impaired subject in the study; the subject reported the IsoTrainer being easier to use.

### Use of MusicGlove as an assessment tool

The MusicGlove game saves quantitative data about user performance to the computer after each song. We found that a simple overall measure of game play success, the percentage of total notes hit during the assessments, strongly correlated with the B & B score, a standard clinical assessment. Further, we found a linear correlation between B & B score for both the Dexterity test and Speed test for both percentage of total notes hit and percentage of notes hit for each grip type. This suggests that the MusicGlove paradigm can be used to provide feedback to both clinicians and users about their progress which is relevant to a variety of activities of daily living; i.e. those that require grip and transport of small objects. Notably, this feedback can be attained as a natural part of therapeutic use of the MusicGlove and does not require separate, complex assessment protocols.

MusicGlove scores were sensitive to within- and between-session gains for total percentage of notes hit and percentage of notes hit per grip type. On average, we found that participants significantly improved in the number of total notes hit and individual notes hit in both the Dexterity test and the Speed test from the start to the end of each session. Overall subjects improved a smaller amount between sessions. A larger within-session improvement may be attributable to short-term motor learning that produces an acute improvement in finger dexterity.

Scores from the MusicGlove game can also provide insight into hand motor impairment mechanisms, as some grips were consistently more impaired. Participants performed sequentially worse at the Speed test in frets 1–5 (fret 1 being a key pinch grip and fret 5 being a pincer grip with the little finger). Note that there may be an order effect due to the ordered sequence of notes on the Speed test. The order unlikely has a significant effect as the sequence is reversed for half of the test. This finding is supported by the grip strength test, where participants performed sequentially worse with pincer grips with middle, ring and little fingers.

## Conclusions

The results of this study showed that after six 45 minute sessions, participants improved the ability to grip small objects more using the MusicGlove paradigm compared to conventional hand exercises, a result that may be attributable to the many repetitions of thumb and finger movements and higher engagement and motivation. When we removed some of the sensory input by making the music-based training isometric (i.e. we reduced the dynamic nature of the proprioceptive input), we found a trend towards decreased effectiveness, and a strong preference of participants toward the dynamic training. A simple measurement of success in the MusicGlove game, % of notes hit, was sensitive to changes within and between therapy sessions, and was clinically valid in the sense that it correlated strongly with the Box and Block score. This measure also provides insight into the increased level of impairment associated with movement of fingers 3–5. We expect that the% of notes hit measure will be valuable to users of the MusicGlove and their clinical caregivers to monitor their progress in improving hand function. Future research will test the feasibility of using this device in a domicile setting with individuals with subacute and chronic stroke, and other populations that exhibit hand impairment.

## Competing interests

Nizan Friedman, David Reinkensmeyer, and Mark Bachman are co-founders of Flint Rehabilitation Devices, a company that is commercializing the MusicGlove. David Reinkensmeyer has received payment for consulting and holds equity in Hocoma, a manufacturer of rehabilitation technology. The terms of these interests have been reviewed by the U.C. Irvine Conflict of Interest committee.

## Authors’ contributions

NF led the conception, design, and development of the MusicGlove and IsoTrainer. He also helped run the experimental protocol and helped to draft the manuscript. DJR contributed to the conception and design of the MusicGlove and design of the experimental protocol, and helped to draft the manuscript. GZ was responsible for software and hardware development of the MusicGlove. MB contributed to the conception and design of the MusicGlove and IsoTrainer device and helped draft the manuscript. VC and ANR carried out the experimental protocol. All authors read and approved the final manuscript.

## Supplementary Material

Additional file 1 **The administered table-top exercises guided by a trained physical therapist are shown.** Training with these exercises was compared to training with the MusicGlove and IsoTrainer. Click here for file
