# Supplementary material for: Retraining and assessing hand movement after stroke using the MusicGlove: comparison with conventional hand therapy and isometric grip training
Source: J Neuroeng Rehabil. 2014 Apr 30;11:76. doi: 10.1186/1743-0003-11-76 (PMC4022276; doi:10.1186/1743-0003-11-76)
Supplement: Additional file 1 — The administered table-top exercises guided by a trained physical therapist are shown. Training with these exercises was compared to training with the MusicGlove and IsoTrainer. [file 1743-0003-11-76-S1.pdf]

Finger Passive Range of Motion

Do these exercises X times a day.

Hold for 5 seconds. Relax. Repeat each exercise 10 times.

Flexion

Use your other hand to gently bend _____ finger at large knuckle.

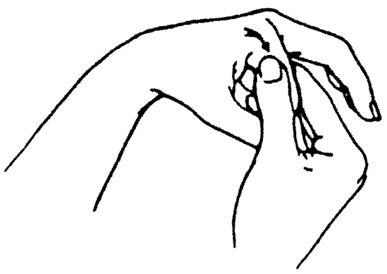

PIP / DIP Composite Flexion

Use your other hand to bend the middle and tip joints of _____ finger.

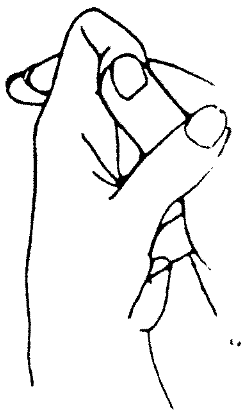

PIP Flexion

Use your other hand to bend the middle joint of _____ finger down as far as possible.

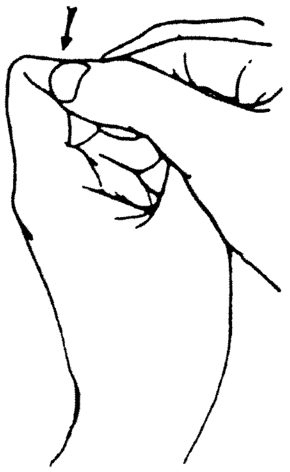

MP / PIP / DIP Composite Flexion

Use your other hand to bend _____ finger at all three joints.

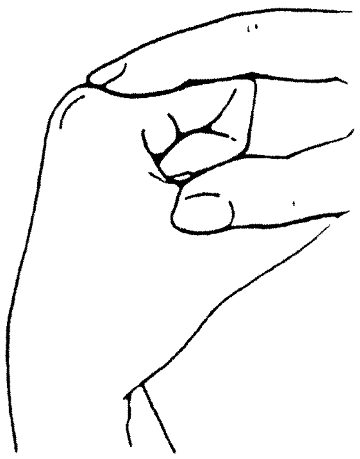

DIP Flexion

Use your other hand to gently bend the tip joint of ________ finger.

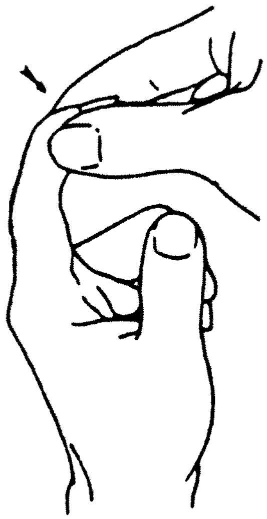

Hand and Finger Exercises

❑ Place your palm flat on a table. Raise and lower your fingers one by one.

Repeat 10 times for X seconds.

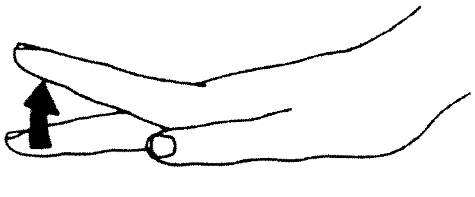

❑ Pick up objects with your hand. Start out with larger objects. (Cup → phone / TV Remote → Pen)

Repeat 10 times for X seconds.

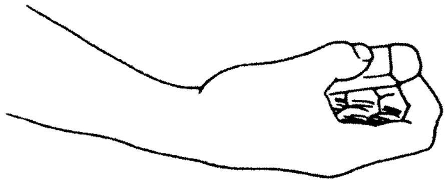

❑ Crumble a piece of paper or cloth into a small ball.

Repeat 10 times for X seconds.

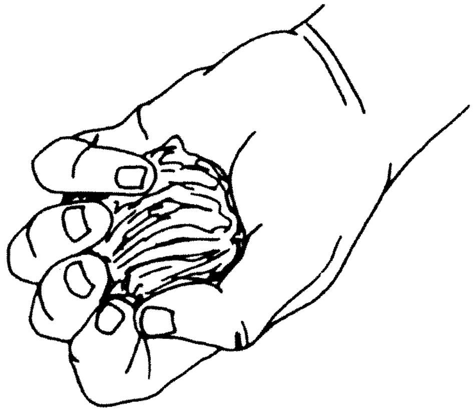

❑ Rest your hand on a table. Spread your fingers wide apart, then bring them back together.

Repeat 10 times for X seconds.

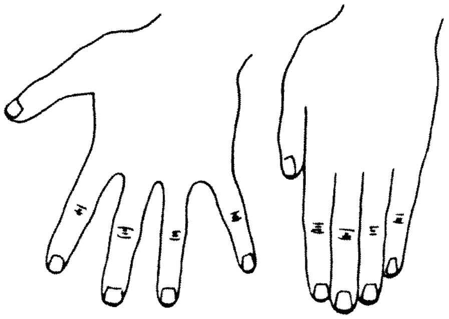

- [ ] Make an "O" by touching your thumb to each fingertip.

  Repeat 10 times for X seconds.

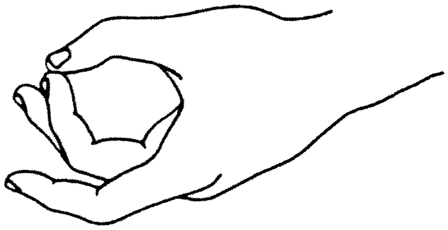

- [ ] Bend the end joint of your finger, keeping the base and middle joints straight. Hold this position. Relax and then straighten the end joint. Hold this position.

  Repeat 10 times for X seconds.

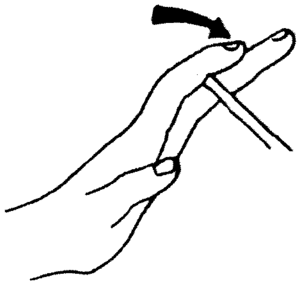

- [ ] Bring the fingertips in tightly to the top of the palm of your hand. Keep your first knuckles straight. Open fingers fully.

  Repeat 10 times for X seconds.

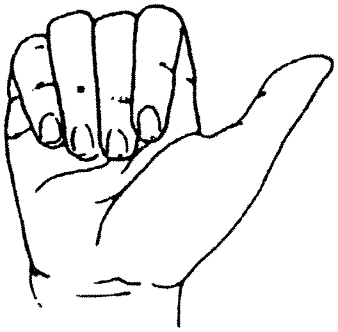

- [ ] Bend your thumb toward the base of your little finger. Spread the thumb away from the index finger.

  Repeat 10 times for X seconds.

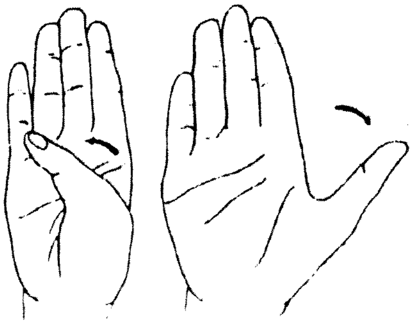

Six Pack Active Hand Exercises

Repeat these exercises 10 times, for X times a day.

1. Make a tabletop with your fingers by keeping your wrists and your fingers straight. Pull fingers down to the top of the palm, by bending at the knuckles.

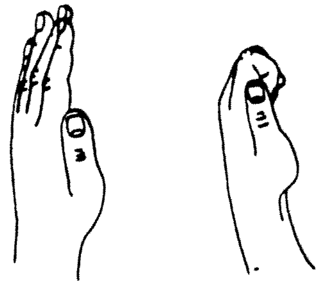

2. Keep your knuckles and wrist straight. Bend and straighten your fingers.

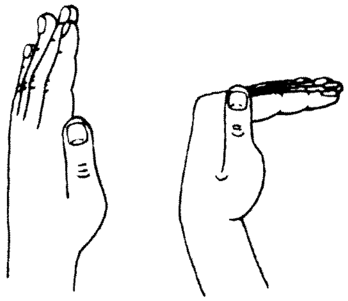

3. Make a fist, being sure each joint is bending as much as possible.

4. Straighten your fingers as much as possible.

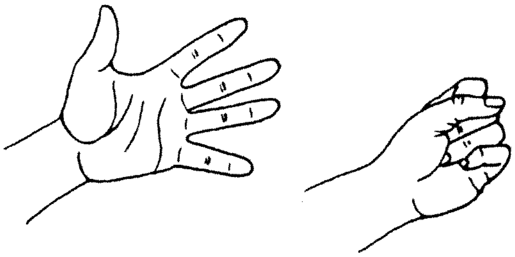

Passive Wrist Exercises

Do these exercises X times a day.
Hold for 5 seconds. Relax. Repeat each exercise 10 times.

Extension

Lift your hand at the wrist and push back as far as you can, using your other hand.

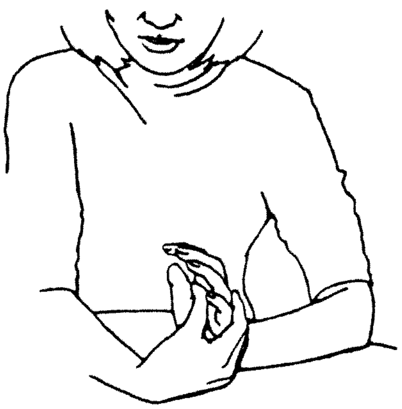

Radial Deviation

Put your wrist and palm on the table. Place your other hand on top to keep it steady while you bring the elbow **inward**.

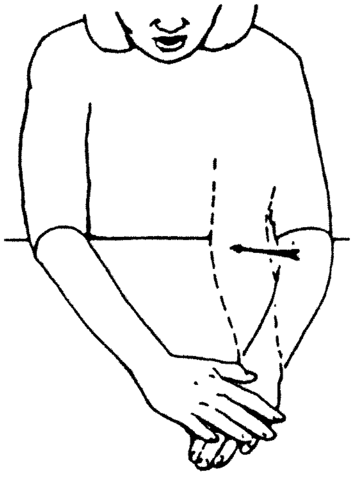

Extension 2

- Keep your palm on the table. Use your other hand on top to help lift the elbow upward.
- Rest your hand with your palm on your hip. Move your elbow out at your side.

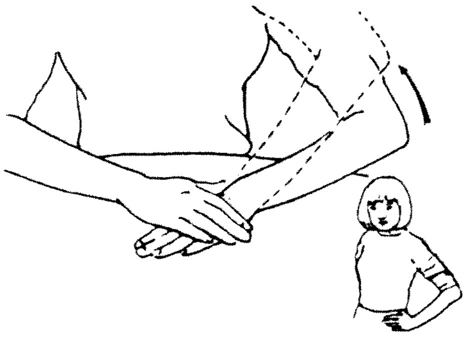

Isometric Wrist Exercises

Do these exercises X times a day.
Hold for 5 seconds. Relax. Repeat each exercise 10 times.

Extension

Keep your forearm steady in a palm down position. Use your opposite hand to push against an upward movement of your hand at the wrist.

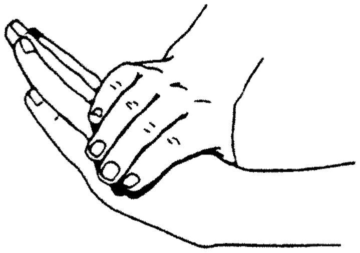

Ulnar Deviation

Hold your forearm steady with the thumb up. Use your other hand to push against a downward movement at the wrist.

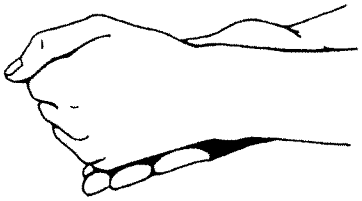

Flexion

Hold your forearm steady with the palm up. Use your opposite hand to push against an upward movement of your hand at the wrist.

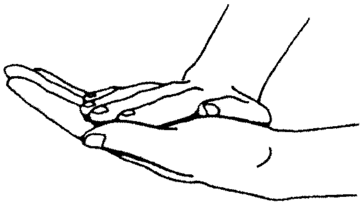

Radial Deviation

Hold your forearm steady in a thumb up position. Use your other hand to push against an upward movement at the wrist.

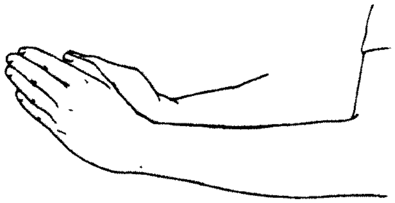

Ulnar Deviation

Put your palm and wrist on the table. Place your other hand on top to keep it steady while you bring the elbow **outward**.

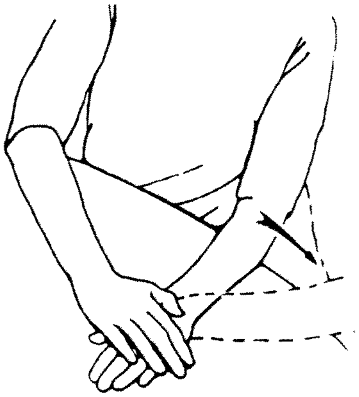

Flexion

Rest your palm at the edge of a table. Hold it steady with your other hand on top. Bring your elbow downward.

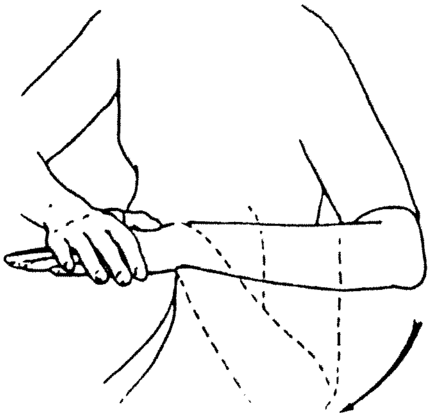

Active Range Of Motion Exercises: Wrists

Repeat these exercises __10__ times for __X__ times a day.

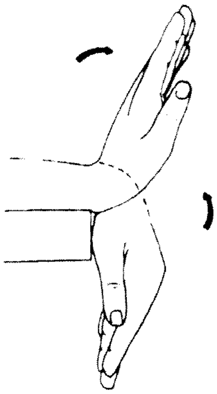

❑ Rest your forearm firmly on a table top and hang your hand over the edge of the table. Bend your wrist up and down as far as possible.

❑ Rest your forearm firmly on table top and hang your wrist over the edge of the table. Move your wrist in circles to the right and to the left.

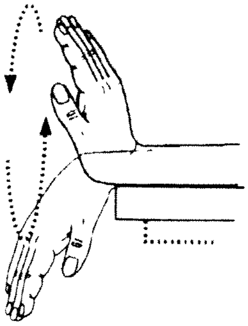

❑ Put your forearm with your hand, palm down, on the table. Move your hand toward the little finger side. Then move the hand toward the thumb side. Keep your forearm still.

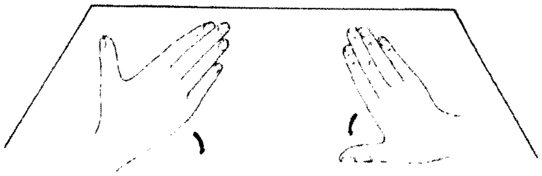

❑ Place your arms at your side with elbows bent. Turn your hand so that the palm faces up to the ceiling. Now turn your hand so that the palm faces down to the floor, keeping your elbow tucked in at your side.

Repeat _____ times for _____ seconds.

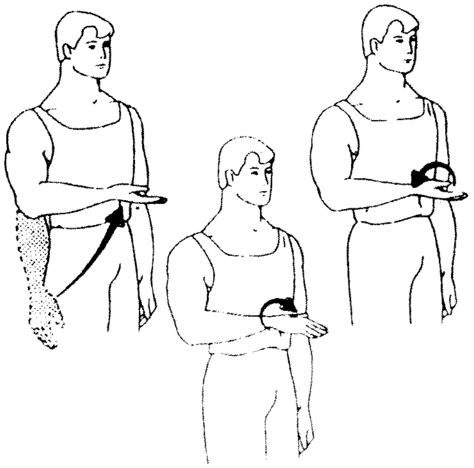

University of California, Irvine Healthcare
Occupational Therapy Department

Active Hand Exercises

Perform these exercises______ repetitions, ______ times a day. Remember to stop if pain occurs with these activities/exercises.

The purpose of these exercises is not only to increase the movement in your fingers, but also to improve your coordination and dexterity.

- Place your palm flat on a table, raise and lower your fingers one by one.
- Make an "O" by touching each fingertip to your thumb one at a time.
- Crumble a sheet of newspaper into a small ball with one hand, palm down.
- Pick up coins or buttons of assorted sizes, alternating fingers.
- With your hand resting on a table, spread fingers wide and then bring them together.

  Bend each finger down, then all the way up at all three joints, making sure to support the joint below the one you're bending.
- Turn empty spools of thread in your hand with your fingertips.
- Pick up handfuls of sand, rice or popcorn and let it trickle through your fingers.
- Set up dominos on their sides.
- Pick up a pencil and roll it between your thumb and fingers.
- Shuffle playing cards.
- Hold cards in your non-affected hand, and turn cards over as quickly as you can with your affected hand.
- Place cards on the table in front of you and turn the cards over as quickly as you can *alternate* hands.
- Divide a deck of cards into two piles and place them on a table. Turn the cards over using both hands simultaneously.
